# Synthesis, Characterization, and Evaluation of Silver Nanoparticle-Loaded Carboxymethyl Chitosan with Sulfobetaine Methacrylate Hydrogel Nanocomposites for Biomedical Applications

**DOI:** 10.3390/polym16111513

**Published:** 2024-05-27

**Authors:** Sonaimuthu Mohandoss, Kuppu Sakthi Velu, Salim Manoharadas, Naushad Ahmad, Subramanian Palanisamy, SangGuan You, Muhammad Saeed Akhtar, Yong Rok Lee

**Affiliations:** 1 School of Chemical Engineering, Yeungnam University, Gyeongsan 38541, Republic of Korea; sakthi.velu4@gmail.com (K.S.V.); passions.malik@gmail.com (M.S.A.); 2 Department of Botany and Microbiology, College of Science, King Saud University, Riyadh 11451, Saudi Arabia; smanoharadas@ksu.edu.sa; 3 Department of Chemistry, College of Science, King Saud University, Riyadh 11451, Saudi Arabia; anaushad@ksu.edu.sa; 4 East Coast Life Sciences Institute, Gangneung-Wonju National University, Gangneung 25457, Republic of Korea; spalanisamy33@gwnu.ac.kr (S.P.); sangguanyou@gmail.com (S.Y.)

**Keywords:** AgNPs, hydrogels, nanocomposites, release, antimicrobial, anticancer

## Abstract

In this study, nanocomposites of AgNPs encapsulated in carboxymethyl chitosan (CMCS) with sulfobetaine methacrylate (SB) hydrogel (AgNPs/CMCS-SB) were synthesized. The UV-Vis spectra indicated the presence of AgNPs, with a broad peak at around 424 nm, while the AgNPs-loaded CMCS-SB nanocomposite exhibited absorption peaks at 445 nm. The size and dispersion of AgNPs varied with the concentration of the AgNO_3_ solution, affecting swelling rates: 148.37 ± 15.63%, 172.26 ± 18.14%, and 159.17 ± 16.59% for 1.0 mM, 3.0 mM, and 5.0 mM AgNPs/CMCS-SB, respectively. Additionally, water absorption capacity increased with AgNPs content, peaking at 11.04 ± 0.54% for the 3.0 mM AgNPs/CMCS-SB nanocomposite. Silver release from the nanocomposite was influenced by AgNO_3_ concentration, showing rapid initial release followed by a slower rate over time for the 3.0 mM AgNPs/CMCS-SB. XRD patterns affirmed the presence of AgNPs, showcasing characteristic peaks indicative of a face-centered cubic (fcc) structure. The FTIR spectra highlighted interactions between AgNPs and CMCS-SB, with noticeable shifts in characteristic bands. In addition, SEM and TEM images validated spherical AgNPs within the CMCS-SB hydrogel network, averaging approximately 70 and 30 nm in diameter, respectively. The nanocomposite exhibited significant antibacterial activity against *S. aureus* and *E. coli*, with inhibition rates of 98.9 ± 0.21% and 99.2 ± 0.14%, respectively, for the 3.0 mM AgNPs/CMCS-SB nanocomposite. Moreover, cytotoxicity assays showcased the efficacy of AgNPs/CMCS-SB against human colorectal cancer cells (HCT-116 cells), with the strongest cytotoxicity (61.7 ± 4.3%) at 100 μg/mL. These results suggest the synthesized AgNPs/CMCS-SB nanocomposites possess promising attributes for various biomedical applications, including antimicrobial and anticancer activities, positioning them as compelling candidates for further advancement in biomedicine.

## 1. Introduction

The integration of silver nanoparticles (AgNPs) with hydrogel matrices has recently gained considerable attention in biomedical research owing to the unique antimicrobial properties of AgNPs and the versatile characteristics of hydrogels for drug delivery and tissue engineering applications [1]. Among these, silver nanoparticles (AgNPs) have emerged as a particularly intriguing class of nanomaterials owing to their remarkable physical, chemical, and biological properties. The distinctive characteristics of AgNPs render them promising candidates for a wide range of applications across various sectors, including biomedical, environmental, and industrial fields [2]. The AgNPs exhibit exceptional antimicrobial activity attributed to their high surface area-to-volume ratio, which enhances contact with microbial membranes, leading to disruption of cellular integrity and inhibition of microbial growth [3]. Furthermore, AgNPs have demonstrated promising anticancer activity by inducing apoptosis and inhibiting proliferation in cancer cells, thus making them potential candidates for cancer therapy [4,5]. In biomedical applications, AgNPs have shown immense potential as antimicrobial agents, drug delivery vehicles, and diagnostic tools. Their ability to efficiently penetrate bacterial cell membranes and disrupt cellular processes has led to their exploration as alternatives to conventional antibiotics, particularly considering the rising antibiotic resistance. Furthermore, the surface functionalization of AgNPs enables the targeted delivery of therapeutic agents to specific sites within the body, reducing systemic toxicity and enhancing therapeutic efficacy [6]. Despite the notable progress in understanding and exploiting the properties of AgNPs, several challenges remain, such as the nanoparticle stability, cytotoxicity, environmental impact, and scalability of the synthesis methods that facilitate the widespread adoption of AgNPs in practical applications. Moreover, the complex interactions of AgNPs with biological systems and the environment necessitate comprehensive studies to assess their safety and environmental impact.

Hydrogels have gained significant attention in biomedical research and applications owing to their unique properties, including a high water content, biocompatibility, and tunable mechanical and chemical characteristics [7]. Among the diverse range of hydrogel-forming polymers, carboxymethyl chitosan (CMCS) is a notable and promising candidate for biomedical applications owing to its biocompatibility, biodegradability, and functional groups that can be modified to tailor its properties for specific applications [8,9]. Chitosan, which is a natural polysaccharide derived from chitin, is inherently biocompatible and has antimicrobial properties, making it an attractive material for biomedical applications [10,11]. However, the poor solubility of chitosan in physiological conditions limits its utilization, particularly in aqueous environments. Carboxymethylation of chitosan introduces carboxyl groups onto the polymer backbone, enhancing its solubility and allowing for the facile manipulation of its physicochemical properties [12]. The resulting CMCS exhibits improved water solubility, biocompatibility, and mucoadhesive properties, making it suitable for various biomedical applications, including drug delivery, tissue engineering, wound healing, and regenerative medicine [13]. Furthermore, the presence of carboxyl groups in CMCS facilitates further chemical modifications, such as crosslinking and grafting, to impart the desired functionalities and enhance its performance in specific applications. Hydrogels based on CMCS offer several advantages for biomedical applications. Owing to their hydrophilic nature and porous structure, CMCS hydrogels can absorb and retain large amounts of water and bioactive molecules, making them ideal for drug delivery, antibacterial, and anticancer systems [14]. Moreover, the mechanical properties and degradation kinetics of CMCS hydrogels can be tailored to match the requirements of different tissue engineering and regenerative medicine applications. Incorporating bioactive agents, such as growth factors, drugs, and nanoparticles, into CMCS hydrogels further expands their functionality and therapeutic potential [11]. The controlled release of bioactive molecules from CMCS hydrogels can be achieved via diffusion, degradation, or stimuli-responsive mechanisms, enabling spatiotemporal control over therapeutic delivery as well as promoting tissue regeneration and wound healing.

Sulfobetaine methacrylate (SB) is a zwitterionic polymer known for its exceptional antifouling properties and low immunogenicity; thus, it is ideal for enhancing the biocompatibility and non-fouling behavior of hydrogel matrices [15,16]. Incorporating CMCS and SB into hydrogel matrices offers numerous advantages for biomedical applications [17]. First, the synergistic interaction between CMCS and SB enhances the mechanical properties and stability of hydrogels, making them suitable for load-bearing applications in biomedical engineering [18]. Second, the non-fouling properties of SB prevent protein adsorption and cell adhesion on hydrogel surfaces, reducing the risk of inflammatory responses and improving biocompatibility [19]. Third, the stimuli-responsive behavior of CMCS-SB hydrogels enables the controlled release of encapsulated drugs or bioactive molecules in response to external triggers, facilitating spatiotemporal control over therapeutic delivery [20]. Combining CMCS and SB in hydrogel matrices demonstrates significant potential for various biomedical applications [11,18]. In drug delivery systems, CMCS-SB hydrogels offer improved drug-loading capacity, prolonged circulation times, and targeted delivery, leading to enhanced therapeutic efficacy and reduced side effects [18,19,21,22]. In tissue engineering, CMCS-SB hydrogels provide a conducive microenvironment for cell growth, proliferation, and differentiation, promoting tissue regeneration and integration. When combined with CMCS and AgNPs in hydrogel matrices, SB further improves stability, reduces protein adsorption, and enhances responsiveness to environmental stimuli, such as pH, temperature, or ionic strength [23,24]. The synergistic effects of AgNPs, CMCS, and SB hydrogels can lead to enhanced antibacterial and anticancer activities, offering new opportunities for the development of therapeutic agents with improved efficacy and reduced side effects. In this study, we aim to synthesize and characterize silver nanoparticle-loaded carboxymethyl chitosan with sulfobetaine methacrylate hydrogel (AgNPs/CMCS-SB) nanocomposites and evaluate their potential in antibacterial and anticancer applications.

## 2. Materials and Methods

### 2.1. Materials

Carboxymethyl chitosan (Carboxymethylation >/= 80%; CMCS), poly (ethylene glycol)-block-Poly (sulfobetaine methacrylate; SB) (PEG average M_n_ 5000, PSBMA M_n_ 13,000), silver nitrate (AgNO_3_; ≥99.0%), sodium borohydride (NaBH_4_; ≥98.0%), polyvinylpyrrolidone (MW 40,000; PVP), and ammonium persulfate ((NH_4_)_2_S_2_O_8_; ≥98.0%) were all purchased from Sigma-Aldrich, Seoul, Republic of Korea. The water used in the experiment underwent ultra-purification and was generated by the Milli-Q system (Milpitas, CA, USA). All additional reagents were of analytical grade and did not necessitate further purification prior to usage. 

### 2.2. Synthesis of AgNPs

The synthesis of AgNPs via chemical reduction involves several steps [25]. Initially, a solution of AgNO_3_ is prepared by dissolving a specified quantity of AgNO_3_ in 100 mL of distilled water to attain a concentration of 1 mM. Concurrently, a solution of 100 mM NaBH_4_ is prepared by dissolving it in distilled water (1.0 mL). The AgNO_3_ solution is then heated to a predetermined temperature of 80 °C in a clean reaction vessel, with constant stirring to ensure even heating. Gradually, the 100 mM NaBH_4_ solution is added drop by drop to the heated AgNO_3_ solution while stirring continuously, instigating the reduction in silver ions to atoms, which subsequently aggregate to form AgNPs, leading to a color transition of the solution from colorless to yellowish-brown. To stabilize the formed AgNPs and prevent aggregation, 0.10 g of PVP is introduced into the reaction mixture. Upon completion of the reaction, the synthesized AgNPs can be characterized using various analytical techniques.

### 2.3. Synthesis of AgNPs-Loaded CMCS-SB Hydrogel

The synthesis of the CMCS-SB copolymer comprises several sequential steps aimed at grafting sulfobetaine methacrylate (SB) onto carboxymethyl chitosan (CMCS) [26]. Initially, 2.0 g of CMCS is dissolved in 100 mL of a 1 vol% acetic acid aqueous solution. The mixture is then heated to 65 °C while maintaining constant stirring at 500 rpm throughout the reaction. Following this, 0.2 g of (NH_4_)_2_S_2_O_8_ is added to the CMCS solution, and N_2_ gas is bubbled through it for 30 min, creating an oxygen-free environment crucial for the polymerization reaction. Meanwhile, SB is dissolved in deionized water, and this solution is gradually incorporated into the reaction system. The reaction continues for 6 h at a consistent temperature and stirring rate. Upon completion, the solution is cooled, followed by a 72-h dialysis process using a dialysis bag with a molecular cutoff weight ranging from 8000 to 14,000, aiming to eliminate small molecules, salts, and unreacted monomers. Finally, the solution undergoes freeze-drying to yield the CMCS-SB hydrogels in a dry form ready for collection and subsequent utilization in various applications.

To prepare the AgNPs-loaded CMCS-SB hydrogels, the synthesized AgNPs are incorporated into the CMCS-SB hydrogel matrix during the gelation process [27]. Initially, different concentrations of AgNO_3_ (1.0, 3.0, and 5.0 mM), synthesized through chemical reduction, are dispersed in the CMCS-SB solution before gelation. This dispersion is achieved by thoroughly mixing in a water bath at 90 °C to ensure the even distribution of AgNPs within the CMCS-SB hydrogel matrix solution. Following this, the gelation process is initiated by adjusting the pH of the CMCS-SB solution to 6, depending on the specific gelation mechanism employed. Upon completion of gelation, the AgNPs-loaded CMCS-SB hydrogel forms, ready for further characterization and utilization across various applications.

### 2.4. Characterizations

The UV-vis spectra of the AgNPs, CMCS-SB hydrogel, and AgNPs/CMCS-SB nanocomposites with varying irradiation times were obtained by a UV-vis spectrophotometer (UV 3220-Optizen, Daejeon, Republic of Korea) in the wavelength range of 200–800 nm. The XRD patterns of the AgNPs, CMCS-SB hydrogel, and AgNPs/CMCS-SB nanocomposites were obtained using a Cu Kα radiation source (k = 1.54064 Å) generated by an (PANalytic X’Pert Philips, MRD model, Tokyo, Japan) X-ray diffractometer operating at a voltage and current of 40 kV and 40 mA, respectively. To assess the chemical composition of the AgNPs, CMCS-SB hydrogel, and AgNPs/CMCS-SB nanocomposites, FTIR analysis was conducted by employing a spectrometer (Perkin Elmer, Shelton, CT, USA) with a resolution of 4 cm^−1^, utilizing an attenuated total reflection (ATR) in the wavenumber range of 4000–400 cm^−1^. The surface morphology of the materials was examined via SEM (JEOL JSM-6490LA, Tokyo, Japan), whereas the internal structural morphologies of the AgNPs and AgNPs/CMCS-SB nanocomposites were characterized via TEM (200 kV, JEOLJEM-2100, Tokyo, Japan). For the SEM analysis, the samples were prepared by casting 0.1 mg/mL of water suspensions onto a silicon substrate and allowing them to dry prior to examination.

### 2.5. Swelling and Water Absorption

The swelling, water solubility, and water absorption of the CMCS-SB hydrogels and AgNPs-loaded CMCS-SB hydrogels were assessed via a gravimetric approach. Initially, both types of hydrogels were cut into 3 cm × 3 cm. These segments were then subjected to drying in an oven at 45 °C until a constant weight (Wd) was achieved. Subsequently, the first set of segments was immersed in 30 mL of PBS buffer solution with a pH of 7.4 for 24 h. After removal from the solution, any excess surface liquid was carefully removed using filter paper, followed by reweighing (W_ts_) the segments. The swelling of both types of hydrogels was determined using Equation (1) as follows:

Swelling degree (%) = (W_ts_ − W_td_)/W_td_ × 100 (1)

Subsequently, the swollen CMCS-SB hydrogels and AgNPs-loaded CMCS-SB hydrogels were subjected to drying in an oven at 45 °C until a constant weight (W_t1_) was achieved. The water solubility of both types of hydrogels was then determined using Equation (2) as follows:

Water solubility (%) = (W_td_ − W_t1_)/W_td_ × 100 (2)

The remaining groups of the CMCS-SB hydrogels and AgNPs-loaded CMCS-SB hydrogels were exposed to 25 °C and a relative humidity of 50% for 24 h, after which they were weighed (W_t2_). The water absorption of the films was then determined using Equation (3) as follows:

Water absorption (%) = (W_t2_ − W_td_)/W_td_ × 100 (3)

### 2.6. Release of Silver from AgNPs/CMCS-SB Nanocomposites

The release profiles of silver from the AgNPs/CMCS-SB nanocomposites were obtained by monitoring the optical density (O.D.) at various time intervals. Specifically, approximately 1.0 g of the AgNPs/CMCS-SB nanocomposites were placed in a flask containing 10 mL of water at 37 °C. The flask was then placed on a rotary shaker and oscillated at a frequency of 60 rpm. The amount of silver released was quantified by measuring the O.D. at its peak wavelength (420 nm) using a UV-vis spectrophotometer (UV 3220-Optizen, Daejeon, Republic of Korea).

### 2.7. Antibacterial Study

The antibacterial efficacy of the prepared nanocomposites (CMCS-SB hydrogel, AgNPs, and AgNPs/CMCS-SB nanocomposite) against *S. aureus* and *E. coli* was evaluated using methods adapted from Yalei Liu et al., 2022 [27]. Initially, 0.5 g of each material was prepared in sterile test tubes. Then, 20 μL of the bacterial suspension (OD_600_ = 0.1) was added to the surface of each material in separate test tubes. After inoculating at 37 °C for 2 h, 980 μL of sterile medium was added to suspend any remaining bacteria. A control group with the same bacterial suspension but without contact with the materials was also prepared. Subsequently, 100 μL of the bacterial suspension from each sample was spread onto agar plates using a sterile coating rod. After incubating at 37 °C for 24 h, bacterial colonies on the plates were identified and counted using ImageJ software. The counting process was repeated six times, and the average was calculated. The antimicrobial performance was quantified using the following Equation (4):

Mortality (%) = blank control group − sample group/blank control group × 100%(4)

### 2.8. In-Vitro Cytotoxicity Study

Initially, HCT-116 cells were seeded in a 96-well microplate containing the cell culture medium (DMEM with 1% penicillin-streptomycin and 10% FBS) at a density of 1 × 10^6^ cells/mL. The plates were incubated at 37 °C under 5% CO_2_ for 24 h. Subsequently, the cells were treated with different concentrations of the CMCS-SB hydrogel, AgNPs, and AgNPs/CMCS-SB nanocomposite for 24 h. Before treatment, the samples were sterilized via UV irradiation and then extracted at a concentration of 10 mg/mL at 37 °C for 24 h. The resulting extract solution was sterilized by filtration and stored for subsequent use. Cells without treatment served as the control. The experiment was performed in triplicate. During the incubation period, the freshly prepared WST-1 solution (20 μL) was added to the cells, followed by an additional 45 min of incubation. The absorbance was then read at 450 nm. The cell viability was assessed using Equation (5) as follows:

Cell viability (%) = Absorbance of test/Absorbance of control × 100(5)

To gain a comprehensive understanding, morphological changes in the HCT-116 cells were examined using fluorescence microscopy. The cells were seeded in a six-well plate at a density of 1 × 10^5^ cells/well and incubated for 24 h. The cells were then treated with the samples for 24 h at 37 °C, after which the cells were washed with 1× PBS and fixed with a methanol: acetic acid solution (3:1, *v*/*v*). Subsequently, a staining procedure using 4′,6-diamidino-2-phenylindole/Propidium iodide (DAPI/PI) was performed to distinguish live and dead cells. The fluorescent dyes (DAPI/PI) enabled visualization using a Nikon Research Inverted Microscope, the ECLIPSE TS2R-C-AL (Tokyo, Japan), allowing for detailed assessments of the cellular morphology.

## 3. Results and Discussion

### 3.1. Optical Properties of AgNPs/CMCS-SB Nanocomposite

**Figure 1:**
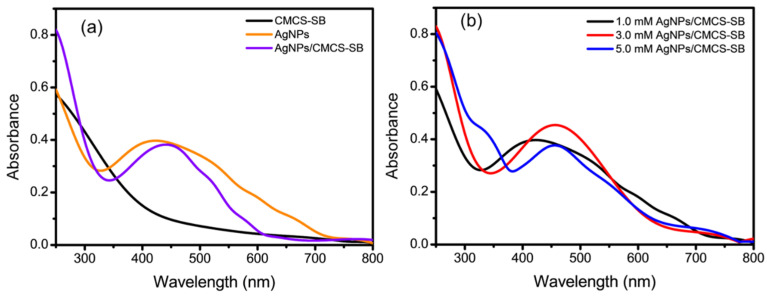
UV-Vis absorption spectra of the (**a**) CMCS-SB hydrogel, AgNPs (1.0 mM), and AgNPs/CMCS-SB nanocomposite, and (**b**) CMCS-SB hydrogel with AgNPs at different concentrations (1.0, 3.0, and 5.0 mM).

The UV-Vis spectra of the AgNPs exhibit a broad peak with a maximum absorption at approximately 424 nm (Figure 1a), corresponding to the Plasmon absorbance characteristic of AgNPs [28]. Conversely, the CMCS-SB hydrogel lacks such a peak owing to its amorphous nature. The interaction between the Ag^+^ ions and functional groups within the polymer network, such as the –OH, –NH_2_, and –C=O groups, facilitates the reduction of Ag^+^ ions under UV irradiation, leading to the formation of AgNPs within the cross-linked polymer matrix [29]. The hydrogel matrix serves as an excellent host for the incorporation of nanoparticles, with the polymer structure controlling the nucleation and growth of AgNPs. Consequently, the presence of AgNPs enhances the overall performance of the AgNPs/CMCS-SB nanocomposite [30]. The apparent color change from violet to dark brown in the AgNPs-loaded CMCS-SB hydrogels coincides with the absorption peaks shifting to approximately 445 nm in the UV-Vis spectra [31]. This shift in the absorption peaks is attributed to the surface plasmon resonance (SPR) phenomenon, which is influenced by the diameter of the larger AgNPs, which exhibit a red-shifted SPR peak at longer wavelengths, whereas nanoparticle aggregates further extend this shift. The formation of AgNPs within the CMCS-SB hydrogels was confirmed via SPR alignment with the typical λ_max_ values of the AgNPs within the visible range of 350–550 nm. The prepared AgNPs/CMCS-SB nanocomposite involves swelling the cross-linked CMCS-SB hydrogels in AgNO_3_ solutions of varying concentrations (1.0, 3.0, and 5.0 mM), as illustrated in Figure 1b. Higher degrees of hydrolysis result in a greater absorption of the Ag^+^ solution by the hydrogel, leading to the formation of larger AgNPs and a broader peak in the UV-Vis spectral region of 350–450 nm [32]. However, as the concentration of the AgNO_3_ solution increases from 3.0 to 5.0 mM, the intensity of the plasmon absorbance decreases, indicating the presence of larger AgNPs and the formation of multi-nanoparticle aggregates. Conversely, in the non-hydrolyzed AgNPs/CMCS-SB nanocomposite system, the shift in peaks is more pronounced, indicating the prevalence of smaller nanoparticles within the matrix [33]. This highlights the influence of hydrolysis on the size distribution and aggregation of AgNPs within the CMCS-SB nanocomposite system.

### 3.2. Swelling and Water Absorption of AgNPs/CMCS-SB Nanocomposite

The swelling, water absorption, and water solubility characteristics of both the prepared CMCS-SB hydrogel and AgNPs/CMCS-SB nanocomposite were precisely evaluated, as summarized in Table 1. Notably, 1.0, 3.0, and 5.0 mM AgNPs/CMCS-SB nanocomposite demonstrated remarkable swelling rates of 148.37 ± 15.63%, 172.26 ± 18.14%, and 159.17 ± 16.59%, respectively [34]. These rates significantly surpassed those of the CMCS-SB hydrogel alone, which exhibited a swelling rate of 138.19 ± 14.83%. The prominent improvement is primarily owing to the graft copolymerization of CMCS and SB, which introduces numerous –COOH, -SO_3_, and quaternary –NH_2_ groups onto the molecular chains [35]. This modification resulted in a substantial increase in the hydration capacity, enhanced the hydrophilicity, and improved the water absorption capabilities. Moreover, the 3.0 mM AgNPs/CMCS-SB nanocomposite exhibited notable improvements in the water absorption and swelling properties compared to both the CMCS-SB hydrogel and other nanocomposite modifications [36]. It can be attributed to the disruption of the -SH bonds between the AgNPs and CMCS-SB, consequently reducing the crystallinity and facilitating the ingress of water molecules. Consequently, the 3.0 mM AgNPs/CMCS-SB nanocomposite demonstrated notably superior water absorption and swelling properties compared to its other counterparts. Additionally, the water solubility of the 3.0 mM AgNPs/CMCS-SB nanocomposite proportionally decreased as the amount of AgNPs added to the CMCS-SB hydrogel increased [37]. Although the CMCS-SB hydrogel exhibited partial dissolution in water at room temperature, incorporating AgNPs improved the hydration ability of the nanocomposite without compromising its water solubility. Consequently, the 3.0 mM AgNPs/CMCS-SB nanocomposite, when employed as a food packaging film, displayed favorable water retention and shape preservation properties, demonstrating its promising potential for such applications.

### 3.3. Release of Silver from Hydrogels

**Figure 2:**
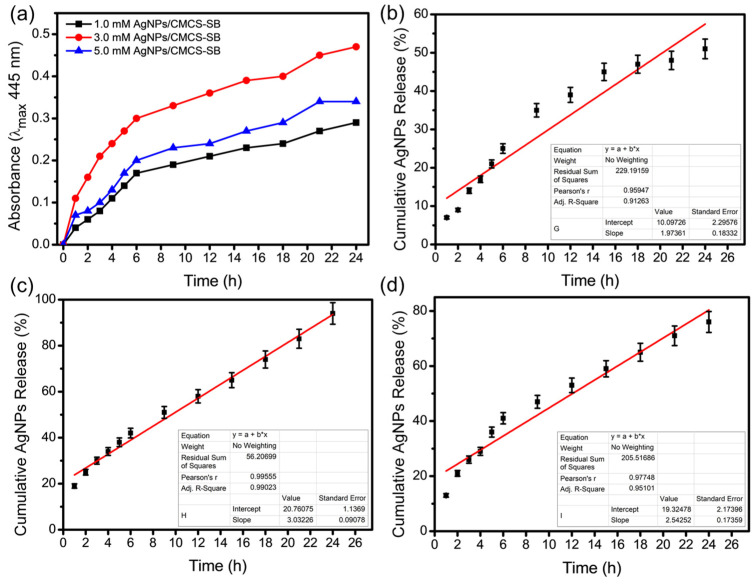
(**a**) Release of silver ions from the AgNPs/CMCS-SB nanocomposite with AgNO_3_ at different concentrations (1.0, 3.0, and 5.0 mM) and Linearity of (**b**) 1.0 mM, (**c**) 3.0 mM, and (**d**) 5.0 mM of AgNPs from the AgNPs/CMCS-SB nanocomposite.

As illustrated in Figure 2a, the 3.0 mM AgNPs/CMCS-SB nanocomposite displayed a release pattern of silver ions characterized by an initial rapid phase followed by a nearly constant release rate [38]. This behavior can be attributed to the heightened hydrophilicity of the matrix associated with an increased AgNO_3_ concentration. Conversely, variations in AgNO_3_ concentration (1.0 and 5.0 mM) resulted in corresponding changes in the initial rate of silver ion release, with a significant increase in release rate observed only after an extended incubation period of approximately 18 h. This prolonged release duration may be attributed to the slower swelling nature of the matrix, observed with both low and high AgNO_3_ contents [39]. Moreover, the linear relationship plotted between various time intervals and AgNPs release from AgNPs/CMCS-SB nanocomposite exhibited a strong linear response within the time range of up to 24 h, with correlation coefficients (R^2^ = 0.9126, 0.9902, and 0.9510) as depicted in Figure 2b–d. Based on these findings, the 3.0 mM AgNPs/CMCS-SB nanocomposite was chosen for further characterization due to its optimal release kinetics. This AgNPs/CMCS-SB nanocomposite strikes a balance between rapid initial release and sustained long-term efficacy, rendering it a promising candidate for subsequent investigations and potential applications in antimicrobial and anticancer formulations.

### 3.4. XDR Pattern of AgNPs/CMCS-SB Nanocomposite

**Figure 3:**
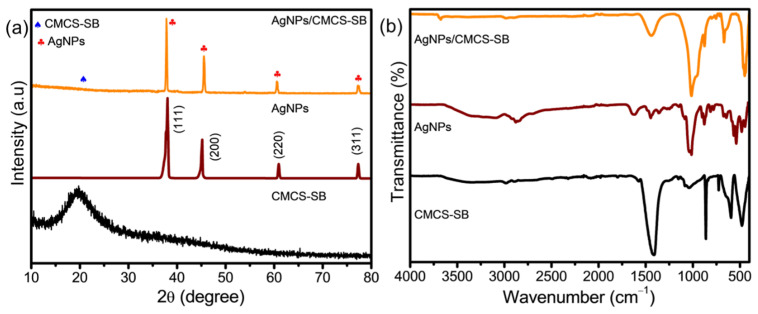
(**a**) XRD pattern and (**b**) FTIR spectra of the CMCS-SB hydrogel, AgNPs, and AgNPs/CMCS-SB nanocomposite.

The crystal structures of the CMCS-SB hydrogel, AgNPs, and AgNPs/CMCS-SB nanocomposite were elucidated via XRD, as illustrated in Figure 3a. The XRD pattern of the CMCS-SB hydrogel displays broad peaks, indicative of its amorphous nature [40]. The XRD pattern of the synthesized AgNPs was analyzed to confirm their identity. Several Bragg reflections with 2θ values of 38.14°, 44.87°, 61.31°, and 77.19° corresponding to the (111), (200), (220), and (311) lattice planes, respectively, were observed [41]. These peaks are characteristic of a face-centered cubic (fcc) structure, commonly associated with metallic silver nanoparticles. Notably, the intensity of the peak corresponding to the (111) plane was higher than that of the other planes. The broadening of these peaks is typical for nano-sized particles, indicating the presence of AgNPs. The XRD pattern of the AgNPs/CMCS-SB nanocomposite also displays diffraction signals at 2θ values of 37.82°, 45.57°, 60.38°, and 77.29°, corresponding to the (111), (200), (220), and (311) diffraction planes of fcc AgNPs [42]. This further confirms the incorporation of AgNPs into the nanocomposite structure. Additionally, the presence of Ag^+^ ions within the network of the nanocomposite was inferred, which can be explained by the incomplete reduction of Ag^+^ ions into Ag^0^ by the borohydride ions. The cross-linked polymer network may have impeded the diffusion of borohydride ions into the CMCS-SB hydrogel network, thereby resulting in this observation.

### 3.5. FTIR Analysis of AgNPs/CMCS-SB Nanocomposite

The FTIR transmission spectra for the prepared CMCS-SB hydrogel, AgNPs, and AgNPs/CMCS-SB nanocomposite are presented in Figure 3b. The FTIR analysis of the CMCS-SB hydrogel reveals distinct absorption peaks corresponding to various functional groups within the molecules. In the FTIR spectrum of SB, the peaks at 1792 and 1599 cm^−1^ correspond to the –C=O group and –CH_3_ stretching vibrations, respectively, within the methyl acrylate structure [43]. Additionally, the peaks at 1154 and 596 cm^−1^ are attributed to the stretching vibrations of the –SO_3_ group and the C-S bond, respectively. Furthermore, a prominent absorption peak at 1427 cm^−1^ was observed, corresponding to the –CH_2_ absorption peak of the quaternary –NH_2_ group in the betaine molecule. A comparison of the infrared spectrum of the graft copolymer CS-SBMA with that of the pure CMCS and SB reveals absorption peaks at 1792, 1599, 1427, 1154, and 596 cm^−1^ in the CS-SBMA hydrogel spectrum. These peaks are associated with the respective functional groups (–C=O, –SO_3_, –NH_2_ groups) present in SB. In the FTIR spectrum of the AgNPs, characteristic peaks were observed at 3365, 2918, 1622, and 1012 cm^−1^, which were attributed to the cyclic –OH, –CH_2_, –C=O, and C–N functional groups, respectively [44]. Changes were observed in the FTIR spectrum of the AgNPs stabilized in the CMCS-SB hydrogel, with the disappearance of absorption bands at 1657 and 1600 cm^−1^ representing the CMCS –CONH_2_ and –NH_2_ groups, and the emergence of a new band at 1677 cm^−1^ indicating the attachment of silver to the nitrogen atom. Additionally, variations in the shape and peak positions of the –NH_2_ and –OH bands at 3647 cm^−1^ occurred owing to their contribution to the reduction and stabilization processes [32,45]. Although there was no strong interaction of the AgNPs with C=O groups in this spectral region, a shift in the band corresponding to the N–H groups of the polymer matrix suggests a weak interaction between the –NH_2_ groups of the polymer chains and AgNPs, stabilizing the nanosystem.

### 3.6. SEM Analysis of AgNPs/CMCS-SB Nanocomposite

**Figure 4:**
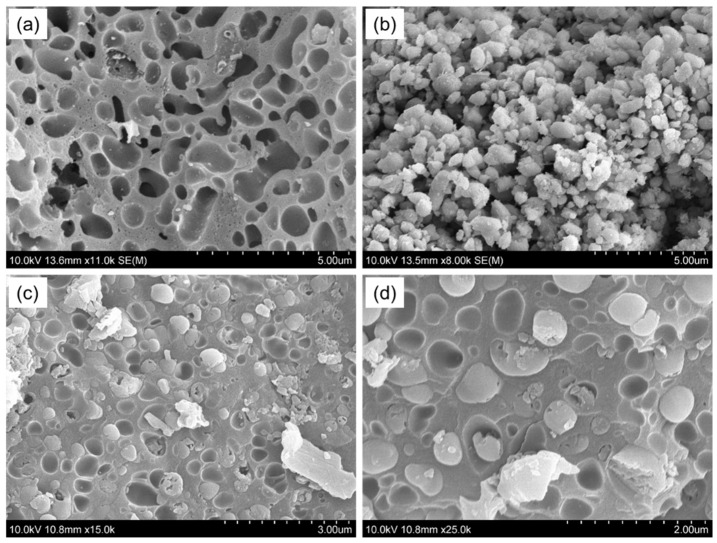
SEM images of the (**a**) CMCS-SB hydrogel, (**b**) AgNPs, and (**c**,**d**) AgNPs/CMCS-SB nanocomposite at different magnifications.

The SEM images depict a smooth surface morphology of the CMCS-SB hydrogel with interconnected pores (Figure 4a). The hydrogel exhibits a porous structure with large, open, and channel-like structures, which indicate regions of water permeation and interaction sites for external stimuli with the hydrophilic groups of the copolymers [46]. Figure 4b presents predominantly spherical AgNPs that are size-dependent [47]. Additionally, to assess the formation of AgNPs within the CMCS-SB hydrogel, SEM images at both lower and higher magnifications are presented (Figure 4c,d). The presence of AgNPs within the CMCS-SB hydrogel networks suggests that when Ag^+^ ions on the surface of the swollen hydrogel are reduced by NaBH_4_, the resulting AgNPs increase the gel porosity, providing a pathway for the reducing agents to enter the bulk of the hydrogel and produce the AgNPs/CMCS-SB nanocomposite [48,49]. Furthermore, incorporating AgNPs into the CMCS-SB hydrogel apparently affects the average pore size, which is likely owing to the interaction of the functional groups with the metallic particles, displacing the water molecules and modifying the structural characteristics of the hydrogel.

### 3.7. TEM Analysis of AgNPs/CMCS-SB Nanocomposite

**Figure 5:**
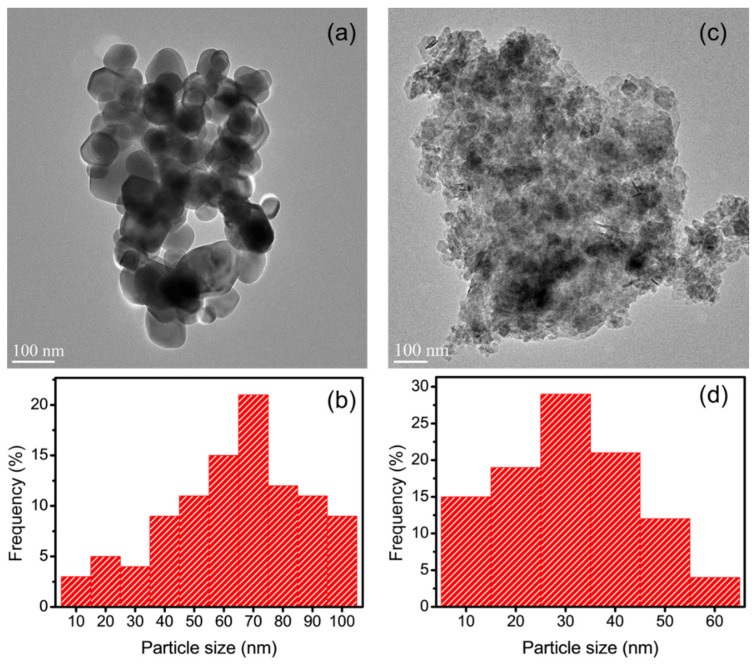
(**a**,**c**) TEM images and (**b**,**d**) histogram of the particle size distribution of the AgNPs and AgNPs/CMCS-SB nanocomposite.

The TEM was utilized to examine the morphology of the prepared AgNPs and AgNPs/CMCS-SB nanocomposite, as depicted in Figure 5. Figure 5a presents the TEM micrograph of AgNPs, revealing spherical nanoparticles with various sizes [50,51]. Figure 5b highlights AgNPs with diameters of approximately 70 nm, which is consistent with the surface plasmon resonance peak observed at 450 nm, indicative of their size distribution. Additionally, TEM images were obtained to observe the presence of AgNPs within the CMCS-SB hydrogel network (Figure 5c) [52,53], for which AgNPs/CMCS-SB nanocomposites were finely ground, equilibrated in distilled water for three days, and then sonicated to facilitate the release of AgNPs from the swollen hydrogel network. This phenomenon can be attributed to factors such as the increased mesh size of the network, the relaxation of polymeric chains entangled around the AgNPs, and decreased binding between the stabilized AgNPs and electron-rich nitrogen atoms of the macromolecular chain. Figure 5d illustrates the AgNPs/CMCS-SB nanocomposites with an average diameter of 30 nm, clearly demonstrating the in-situ formation of AgNPs within the CMCS-SB hydrogel network. Overall, the TEM images confirm the spherical morphology of AgNPs and demonstrate their successful incorporation within the CMCS-SB hydrogel network, validating the effectiveness of the nanocomposite synthesis process.

### 3.8. Antibacterial Properties of AgNPs/CMCS-SB Nanocomposite

**Figure 6:**
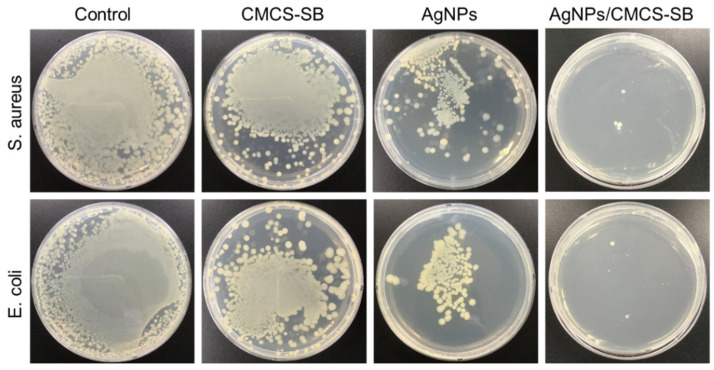
Evaluation of the antibacterial performance of the control, CMCS-SB hydrogel, AgNPs, and AgNPs/CMCS-SB nanocomposite against *E. coli* and *S. aureus*.

The antibacterial properties of the control, CMCS-SB hydrogel, AgNPs, and AgNPs/CMCS-SB nanocomposite were evaluated using the plate counting method against *S. aureus* and *E. coli* bacteria (Figure 6). The bacterial growth on agar plates served as an indicator of the antibacterial activity. The initial antibacterial effect of CMCS likely stems from the presence of amino and carboxyl groups, which can interact with various components on the bacterial surface [8,54], which disrupts cell membrane function, leading to damage or destruction. Although the mechanism of AgNPs is not fully understood, AgNPs are known to release silver ions upon contact with water [55]. These silver ions bind to negatively charged thiol groups (-SH) within the bacterial proteins, thus increasing the membrane permeability and denaturation of the cellular proteins. This process ultimately inhibits bacterial growth and achieves sterilization. Furthermore, the antimicrobial effect of silver ions is long-lasting because they can be reduced back into silver atoms after killing the bacteria. A comparative analysis revealed that although both the AgNPs and CMCS-SB hydrogel exhibited certain antimicrobial properties, incorporating AgNPs into the CMCS-SB hydrogel significantly enhanced its efficacy [36,56]. The superior antibacterial activity of the AgNPs/CMCS-SB nanocomposite, primarily attributed to the presence of AgNPs, was confirmed via experiments on *S. aureus* and *E. coli* bacteria (Figure 6). The AgNPs/CMCS-SB nanocomposite demonstrated significantly improved efficacy against both *S. aureus* (98.9 ± 0.21%) and *E. coli* (99.2 ± 0.14%) compared to the control group, CMCS-SB hydrogel (55.2 ± 0.11% and 57.2 ± 0.17%), and AgNPs (72.8 ± 0.14% and 75.9 ± 0.13%). These findings highlight the superior antibacterial activity of the AgNPs/CMCS-SB nanocomposite, suggesting its potential for use in various biomedical applications.

### 3.9. In-Vitro Cytotoxicity and Imaging of AgNPs/CMCS-SB Nanocomposite

**Figure 7:**
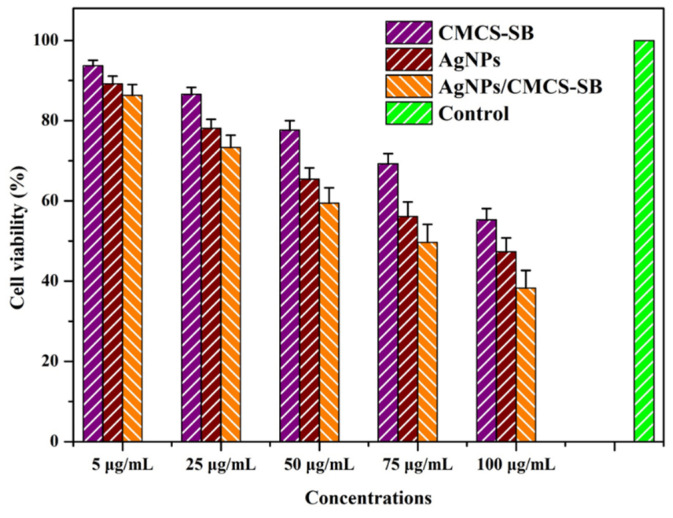
Cell viability of the CMCS-SB hydrogel, AgNPs, and AgNPs/CMCS-SB nanocomposite with HCT-116 cells for 24 h.

In-vitro cytotoxicity tests were conducted to further assess the anticancer activity of the CMCS-SB hydrogel, AgNPs, and AgNPs/CMCS-SB nanocomposite. As shown in Figure 7, HCT-116 cells were co-cultured with the leaching solution of the CMCS-SB hydrogel, AgNPs, and AgNPs/CMCS-SB nanocomposite at various concentrations (5, 25, 50, 75, and 100 μg/mL) for 24 h. The cell survival was assessed using the WST-1 method, revealing a concentration-dependent cytotoxic effect for all the tested samples. Notably, the AgNPs/CMCS-SB nanocomposite exhibited the strongest cytotoxicity (61.7 ± 4.3%) against the HCT-116 cells at 100 μg/mL, suggesting a possible synergistic effect between the AgNPs and CMCS-SB hydrogel [57,58].

**Figure 8:**
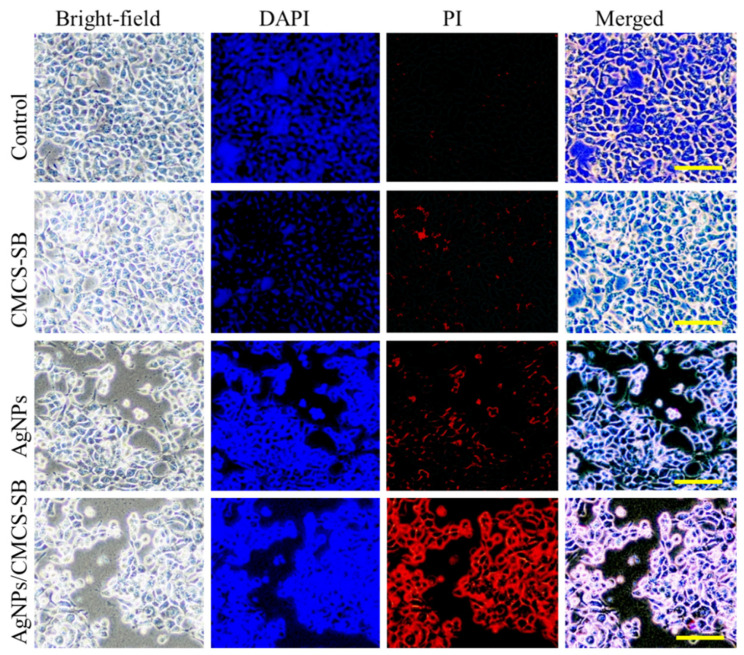
Cell culture effect of the control, CMCS-SB hydrogel, AgNPs, and AgNPs/CMCS-SB nanocomposite with the HCT-116 cells for 24 h on the morphological changes after treatment at 80 μg/mL for 24 h using DAPI/PI with HCT-116 cells. Scale bar~100 μm.

This observation supports the potential application of the AgNPs/CMCS-SB nanocomposite in cancer prevention. Fluorescence microscopy revealed that the CMCS-SB hydrogel and AgNPs alone did not significantly increase the cellular uptake of the material. The combination of CMCS-SB hydrogel and AgNPs significantly increased the cellular uptake, suggesting the effective delivery of the AgNPs/CMCS-SB nanocomposite to cancer cells [59,60]. To better understand this mechanism, HCT-116 cancer cells were incubated with a constant concentration of the CMCS-SB hydrogel, AgNPs, and AgNPs/CMCS-SB nanocomposite (80 μg/mL) for 24 h, followed by DAPI/PI staining to assess the differentiation in cell morphology (Figure 8). The increased presence of PI-stained cells in the AgNPs/CMCS-SB nanocomposite group suggests apoptosis induction. Overall, the results indicated that the AgNPs/CMCS-SB nanocomposite exhibited stronger anticancer activities than those of the CMCS-SB hydrogel and AgNPs alone, suggesting that the CMCS-SB hydrogel in the presence of AgNPs may enhance the cellular uptake efficiency and contribute to enhanced anticancer effects.

Based on these results, the novelty statements for the integration of AgNPs with CMCS-SB hydrogel matrices offer a novel approach in biomedical research combining the unique antimicrobial properties of AgNPs with the versatile characteristics of CMCS-SB hydrogels for drug delivery and tissue engineering applications. The synergistic interaction between CMCS and SB in CMCS-SB hydrogel matrices presents a novel strategy to enhance mechanical properties, stability, and controlled drug release capabilities for biomedical applications. The surface morphology imaging confirms the successful incorporation and distribution of AgNPs within the CMCS-SB hydrogel network, providing novel insights into the synthesis process and nanoparticle morphology, which are vital for understanding the AgNPs/CMCS-SB nanocomposite structure-property relationships. The incorporation of SB into CMCS-SB hydrogel matrices to improve stability, reduce protein adsorption, and enhance responsiveness to environmental stimuli represents a novel advancement in enhancing the biocompatibility and functionality of hydrogel-based systems. The superior antibacterial efficacy of the AgNPs/CMCS-SB nanocomposite against common pathogens such as *S. aureus* and *E. coli*, surpassing that of the hydrogel and AgNPs alone, underscores its novelty as a promising candidate for biomedical applications requiring enhanced antimicrobial properties. The concentration-dependent cytotoxicity of the nanocomposite against HCT-116 cancer cells, along with its ability to induce apoptosis and enhance cellular uptake, presents a novel approach to cancer therapy utilizing hydrogel-based nanocomposites, paving the way for innovative biomedical applications.

## 4. Conclusions

In this study, we successfully synthesized and characterized silver nanoparticle-loaded carboxymethyl chitosan with sulfobetaine methacrylate hydrogel (AgNPs/CMCS-SB) nanocomposites for biomedical applications. The synthesis process resulted in UV-Vis spectroscopy indicating the presence of AgNPs, characterized by a broad peak around 424 nm. Upon integration into the AgNPs/CMCS-SB nanocomposite, AgNPs exhibited absorption peaks at 445 nm in UV-Vis spectra. The size and dispersion of AgNPs varied depending on the concentration of the AgNO_3_ solution, impacting absorbance intensity. Additionally, nanocomposites showed increased swelling rates of 148.37 ± 15.63%, 172.26 ± 18.14%, and 159.17 ± 16.59% for AgNPs/CMCS-SB concentrations of 1.0, 3.0, and 5.0 mM, respectively. The water absorption capacity increased with AgNPs content peaking at 11.04 ± 0.54% for the 3.0 mM AgNPs/CMCS-SB nanocomposite. Conversely, water solubility decreased with increasing AgNPs concentration, with the 3.0 mM AgNPs/CMCS-SB nanocomposite showing the lowest solubility at 10.06 ± 0.37%. Silver release from the nanocomposite depended on AgNO_3_ concentration, notably with the 3.0 mM AgNPs/CMCS-SB exhibiting rapid initial release followed by a slower rate. The XRD patterns confirmed AgNPs in the nanocomposite, displaying characteristic peaks of a face-centered cubic (fcc) structure. In addition, the FTIR spectra suggested interactions between AgNPs and CMCS-SB hydrogel functional groups, with peak shifts indicating silver attachment to nitrogen atoms. Moreover, the SEM and TEM images confirmed the presence of spherical AgNPs within the CMCS-SB hydrogel network with average diameters of approximately 70 and 30 nm, respectively. The AgNPs/CMCS-SB nanocomposite demonstrated potent antibacterial activity against *S. aureus* and *E. coli*, with inhibition rates of 98.9 ± 0.21% and 99.2 ± 0.14%, respectively, for the 3.0 mM AgNPs/CMCS-SB nanocomposite. Notably, AgNPs/CMCS-SB nanocomposite exhibited strong cytotoxicity against HCT-116 cells with fluorescence microscopy, indicating increased intracellular accumulation and suggesting potential for cancer treatment. Compared to conventional polysaccharide-based hydrogel systems, the AgNPs/CMCS-SB nanocomposites exhibited superior swelling rates and water absorption capacities, attributed to the enhanced hydrophilicity and hydration ability conferred by the graft copolymerization of CMCS and SB. The potent antibacterial and anticancer activities demonstrated by the AgNPs/CMCS-SB nanocomposites surpass those of existing polysaccharide hydrogel formulations, underscoring their efficacy in combating microbial infections and cancerous cell proliferation. Further research is warranted to explore their efficacy in vivo and to optimize their formulation for specific therapeutic applications. The multifunctional nature of these nanocomposites is significantly promising for addressing various biomedical challenges and improving patient outcomes in the future.

## Figures and Tables

**Table 1:** Swelling, water absorption, and water solubility of the CMCS-SB hydrogel and AgNPs/CMCS-SB nanocomposite.

Sample	Swelling Rate %	Water Absorption %	Water Solubility %
CMCS-SB	138.19 ± 14.83	9.38 ± 0.72	7.16 ± 0.48
1.0 mM AgNPs/CMCS-SB	148.37 ± 15.63	9.48 ± 0.66	8.99 ± 0.41
3.0 mM AgNPs/CMCS-SB	172.26 ± 18.14	11.04 ± 0.54	10.06 ± 0.37
5.0 mM AgNPs/CMCS-SB	159.17 ± 16.59	10.57 ± 0.33	9.67 ± 0.24

## Data Availability

Data can be requested from the corresponding author due to privacy.
